# The Seed Semipermeable Layer and Its Relation to Seed Quality Assessment in Four Grass Species

**DOI:** 10.3389/fpls.2017.01175

**Published:** 2017-07-04

**Authors:** Yan Y. Lv, Xue Q. He, Xiao W. Hu, Yan R. Wang

**Affiliations:** ^1^The State Key Laboratory of Grassland Agro-ecosystems, College of Pastoral Agriculture Science and Technology, Lanzhou UniversityLanzhou, China; ^2^College of Animal Science and Technology, Northwest A&F UniversityYangling, China

**Keywords:** electrical conductivity, grass species, permeability, seed vigor, semipermeable layer

## Abstract

The existence of a semipermeable layer in grass seeds has been extensively reported, yet knowledge of its influence on tests for seed viability and vigor that depend upon measurement of electrical conductivity (EC) is limited. This study determined the presence and location of the semipermeable layer, and its relation to seed viability and vigor assessment, in seeds of four important grass species-*Elymus nutans* Griseb., *Lolium perenne* L., *Leymus chinensis* (Trin.) Tzvel., and *Avena sativa* L. Intact seeds of *E. nutans, Lolium perenne*, and *Leymus chinensis* exhibited little staining with triphenyl tetrazolium chloride (TTC), and there were no differences in EC between seeds with different germination percentage (GP) (*P* > 0.05). After piercing the seed coat, however, all three species displayed positive staining with TTC, along with a significant negative correlation between EC and GP (*E. nutans*: *R*^2^ = 0.7708; *Lolium perenne*: *R*^2^= 0.8414; *Leymus chinensis*: *R*^2^ = 0.859; *P* < 0.01). In contrast, both intact and pierced seeds of *A. sativa* possessed a permeable seed coat that showed positive staining with TTC and EC values that were significantly negatively correlated with GP [*R*^2^ = 0.9071 (intact) and 0.9597 (pierced); *P* < 0.01]. In commercial seed lots of *A. sativa*, a field emergence test indicated that EC showed a significant negative correlation with field emergence at two sowing dates (*R*^2^= 0.6069, *P* < 0.01 and 0.5316, *P* < 0.05). Analysis of seed coat permeability revealed the presence of a semipermeable layer located in the seed coat adjacent to the endosperm in *E. nutans, Lolium perenne*, and *Leymus chinensis*; however, no semipermeable layer was observed in *A. sativa.* This is the first report of the absence of a semipermeable layer in a grass species. The existence of a semipermeable layer is one of the most important factors affecting seed viability and vigor testing (based on EC measurement) in *E. nutans, Lolium perenne*, and *Leymus chinensis*. Increasing the permeability of the semipermeable layer, e.g., by piercing the seed coat, may permit the use of EC measurement to assess seed vigor in species that possess such a layer.

## Introduction

There are many traits that contribute to seed quality assessment, and seed vigor is the sum total of those properties of the seed that determine its potential activity and performance during germination and seedling emergence (Hampton and TeKrony, 1995). Seed vigor contributes directly to the economic success of commercial crops (Finch-Savage, 1995) and hence seed companies require methods of obtaining a reliable estimate of vigor in order to be able to supply growers with seed lots that possess high vigor. However, seed vigor remains difficult to assess, because it is a complex characteristic that is determined by a number of different factors.

Electrical conductivity (EC) is recommended as a convenient and validated method for assessing seed vigor in *Pisum sativum* L. (Matthews and Bradnock, 1967; Hampton and TeKrony, 1995), and it is widely used for vigor testing of large-seeded legumes, some small-seeded legumes, and *Brassica pekinensis* Rupr. (Hampton and TeKrony, 1995; Wang et al., 2004; Matthews et al., 2009; ISTA, 2016). However, in most grass species, with the exception of *Festuca arundinacea* Keng. (Han et al., 1995), EC values do not change with seed deterioration; examples are *Bromus japonicus* Thunb. ex Murr. (Ching and Schoolcraft, 1968), *Lolium perenne* L. (Hall and Wiesner, 1990), and *Sorghum sudanense* (Piper) Stapf. (Wang, 2003). Previous studies suggested that this phenomenon may be related to the high starch content in the embryo (Yi et al., 1994), the high content of insoluble substances (Han and Mao, 2000), or low EC values of grass seeds due to seed coat semipermeability (Wang, 2003).

A semipermeable layer, defined as a tissue that is permeable to water and gases but that restricts or impedes the exchange of some solutes (Beresniewicz et al., 1995a), exists widely in the seed coats of many species, such as *Allium tuberosum* Rottl. ex Spreng., *Allium cepa* L., *Lycopersicon esculentum* Mill., and *Capsicum annuum* L. (Beresniewicz et al., 1995a), ×*Triticosecale* Wittm. ex A. Camus., *Festuca sinensis* Keng. and *Bromus inermis* L. (Zhou et al., 2013a), *Cucumis sativus* L. (Salanenka and Taylor, 2008; Salanenka et al., 2009), *Lactuca sativa* L. (Hill and Taylor, 1989), and *Sorghum sudanense* (Piper) Stapf.(Yan and Wang, 2008). The presence of a semipermeable layer may limit the exchange of substances between the seed and its external environment, and thus reduce the validity of methods for estimating seed performance based on seed coat permeability. For example, in *Allium tuberosum, Allium cepa, Lycopersicon esculentum*, and *Capsicum annuum* (Beresniewicz et al., 1995a), and in *Sorghum sudanense* (Wang, 2003), the presence of a semipermeable layer results in failure to stain with triphenyl tetrazolium chloride (TTC) unless the layer is punctured. Moreover, the presence of a semipermeable layer has been found to restrict electrolyte leakage in *Sorghum sudanense* (Wang, 2003) and *Cucumis melo* L. (Welbaum and Bradford, 1990), and leakage of amino acids in *Allium tuberosum, Allium cepa, Lycopersicon esculentum*, and *Capsicum annuum* (Taylor et al., 1995). In contrast, *Brassica oleracea* L., which lack a semipermeable layer, showed strong TTC staining and high rates of amino acids leakage in lower vigor seeds (Beresniewicz et al., 1995a; Taylor et al., 1995). These results imply that the presence of a semipermeable layer could affect TTC staining and EC testing, in the latter case by restricting solute leakage. However, although the presence (Hill and Taylor, 1989; Beresniewicz et al., 1995b; Salanenka and Taylor, 2008; Salanenka et al., 2009; Zhou et al., 2013a), location (Beresniewicz et al., 1995b; Yan and Wang, 2008; Zhou et al., 2013a), chemical composition (Beresniewicz et al., 1995a; Zhou et al., 2013a), and formation (Zhou et al., 2013b) of the semipermeable layer has been reported in several species, and been shown to vary between species, its importance in seed quality assessment has rarely been addressed.

Thus, we hypothesized that the presence of a semipermeable layer in grass species may be one of the principal factors that affect TTC staining and EC measurement in the assessment of grass seed quality. In this study, we aimed to determine (1) the presence and location of the semipermeable layer in seeds of four important forage grass species: *Elymus nutans* Griseb., *Lolium perenne* L., *Leymus chinensis* (Trin.) Tzvel., and *Avena sativa* L.; (2) how the semipermeable layer affects staining with TTC and the rate of solute leakage measured by an EC test, thus affecting vigor testing, and (3) whether EC testing can be used to predict seedling emergence when the species concerned does not possess a semipermeable layer.

## Materials and Methods

### Seeds and Seed Lots

The presence and location of the semipermeable layer and seed coat permeability were determined using one seed lot for each species: *E*. *nutans* and *A*. *sativa* were harvested in 2006 from Gannan and Jingtai in Gansu Province, *Lolium perenne* and *Leymus chinensis* were obtained in 2007 from Inner Mongolia, China, and Oregon, United States, respectively. The primary viability for these seed samples was >90%.

A mixture of living and dead seeds was obtained by subjecting seeds to controlled deterioration as described by Hampton and TeKrony (1995). Germination percentage (GP) was 28–96% in *E. nutans*, 0–98% in *Lolium perenne*, 12–94% in *Leymus chinensis*, and 0–95% in *A. sativa*.

An additional 15 commercial seed lots of both *E. nutans* and *A. sativa* were used for field emergence tests on two sowing dates. Each of the seed lots had been harvested between 2011 and 2013 from different regions of Gansu Province, China, and the lots were provided by the Herbage Seed Testing Center of Lanzhou, China. The reported germination of the seed lots ranged from 70 to 82% for *E. nutans* and from 80 to 93% for *A. sativa.* All seed samples were stored dry at 4°C until used.

### Germination Test

Germination percentage was determined in non-treated seed lots and in those subjected to the deterioration treatment. Three replicate groups of 50 seeds were sown in 8-cm Petri dishes on two layers of filter paper moistened with distilled water. Water was added to the dishes as necessary to keep the filter paper moist during the test period. At the end of the germination test, the number of normal seedlings was counted.

### Imbibition and TTC Staining Test of Seeds

Imbibition testing was carried out using three replicates of 100 seeds. Seeds were weighed, soaked in 100 mL of deionized water, and then incubated at 25°C for 24 h. They were then surface-dried and weighed. The imbibition rate was estimated as imbibition (%) = (seed weight after imbibition/initial seed weight) × 100 (Wang et al., 2002).

For TTC staining, intact seeds were soaked in water in a Petri dish for 1 h. They were then stained with 1% (w/v) TTC solution and kept in the dark at 20°C for 36 h. Evaluation of the intensity of staining was performed according to International Seed Testing Association (Hampton and TeKrony, 1995) rules, and the results were divided into three categories: strong staining (seeds completely and well stained), slight staining (partially or completely stained, but faint), and no staining.

Punctured seeds were prepared by piercing the seed coat with a needle to overcome the physical barrier (Hill and Taylor, 1989) and then test imbibition rate and TTC staining was carried out as described above.

### EC Testing

Electrical conductivity testing was performed using a conductivity meter (DST-A, AIP, Tianjin, China) on three replicates of 50 seeds for each sample. Each sample showed a different GP. Seeds were weighed, transferred to 100 mL of distilled water, stirred, covered, and incubated at 20°C for 24 h. Distilled water was used as the control. The EC value (μs cm^-1^ g^-1^) of the incubation solution was measured after 24 h, as described by Hampton and TeKrony (1995). The EC was calculated using the following equation:

$$EC_{\text{t}}\;=\;\left(EC_{\text{s}}-EC_{\text{c}}\right)/M_{\text{s}}$$

where *EC*_t_ is the relative EC, *EC*_s_ denotes the EC of the sample, *EC*_c_ denotes the EC of the control, and *M*_s_ is the total mass of the sample.

Punctured seeds with a range of GPs were prepared as previously described (section “Imbibition and TTC Staining Test of Seeds”), and the EC values were determined as indicated above.

### Field Emergence Test of *E. nutans* and *A. sativa* Commercial Seed Lots

A field emergence test of 15 seed lots was conducted in Xiahe County, in Gansu Province. Seeds were sown in soil by hand, 2–3 cm deep, in a nursery, on two different sowing dates (late April and late May). Each sample consisted of 100 seeds, with four replicates. The seedlings were evaluated after they had emerged from the soil and had grown to 2 cm in height; evaluation continued until no new seedlings emerged.

### Microscopic Analysis of Seed Coat and Endosperm

Fifteen seeds of each species were randomly selected, and the lemma and palea were removed. The seeds were then placed at 20°C in an incubator for 24 h, after which approximately 1 mm^3^ of the embryo surface attached to a small piece of endosperm tissue was removed using a razor blade. The tissue was prepared for semi-thin sections as described by Zheng and Gu (1993). In brief, the embryo tissue was soaked in 3% (w/v) glutaraldehyde solution, and then pre-fixed for 6 h at 4°C before being rinsed three times for 10 min with phosphate buffer solution (PBS, 0.1 M, pH 7.2). The tissue was then fixed overnight in 1% (w/v) osmic acid at 4°C and rinsed three times for 10 min with PBS. The seeds were next dehydrated using an ethanol series (70, 80, 90, and 100% ethanol successively for 15 min each). Ethanol dehydration was repeated twice and was followed by dehydration with epoxypropane. The tissue was then embedded with epoxypropane and Epon812 [1:1 ratio (v/v)] for polymerization (35, 45, and 60°C successively for 24 h each). The tissue was removed from the embedding mold and processed into a block using an ultramicrotome (LKD-2088 – Ultratom V., Bromma, Sweden), and semi-thin sections (2 μm) were cut. Finally, the sections were stained with 0.05% (w/v) toluidine blue, sealed with Canada gum, and observed under a light microscope.

### Transmission Electron Microscopy (TEM) Analysis of the Semipermeable Layer

The degree of lanthanum infiltration can be used to analyze the location of the semipermeable layer in the seed. Fifteen seeds were randomly selected for use in the TEM analysis. First, the lemma and palea were removed and the seeds were incubated at 20°C. Then, individual seeds were soaked for 24 h in deionized water (seeds with a broken seed coat were discarded) and incubated in a 4% (w/v) solution of the tracer compound lanthanum nitrate at 20°C for 24 h. Part of the embryo surface was removed and cut into 1-mm^3^ slices using a razor blade. An embedding block (containing the seed coat together with part of the endosperm) was prepared, and 70-nm-thick sections were cut using an LKD-2088 ultramicrotome. The sections were stained with uranyl acetate for 1.5 h, followed by citrate staining for 7–10 min, and were then subjected to TEM imaging (JEM 1230 – JEOL, Tokyo, Japan). The position of the semipermeable layer was determined by the location of the lanthanum signal.

### Observation of the Semipermeable Layer by Energy-Dispersive X-ray Spectroscopy (EDX)

A sufficient number of seeds, usually 15 seeds, with the lemma and palea removed, were soaked in deionized water at 20°C for 24 h. Individual seeds were then soaked in 4% (w/v) lanthanum nitrate solution at 20°C for 24 h and dried at room temperature. Sections approximately 100-nm thick were then prepared as described previously for TEM. They were then examined using an EDX spectrometer (EDX; United States; KEVE2) and a scanning electron microscope (JSM5600LV; JEOL). The lanthanum spectrum was used to locate the position of the semipermeable layer.

### Statistical Analysis

Linear regression analysis was used to represent the relationship between EC and GP/field emergence, using SPSS 19.0 (IBM Corp., Armonk, NY, United States).

## Results

### Imbibition and TTC Staining

In all four species, both intact and pierced seeds readily imbibed water, but the amount imbibed by pierced seeds was greater than that imbibed by intact seeds (**Table 1**). This indicated that the seed coat does not prevent water uptake. Further, non-aged seeds (intact seeds) germinated normally, indicating that gas exchange is not impaired by the seed coat. The TTC staining test, to evaluate uptake of vital stain into non-aged intact seeds, showed little strong TTC staining in *E. nutans, Lolium perenne*, and *Leymus chinensis* embryos; however, if the seed coats had been pierced, most of the embryos were strongly stained (94, 84, and 89%, respectively). This indicates that in *E. nutans, Lolium perenne*, and *Leymus chinensis* the seed coat prevented penetration of TTC. In contrast to these three species, a high proportion of intact *A. sativa* seeds (79%) showed strong staining, suggesting that the seed coat in this species was permeable to TTC; piercing the seed coat resulted in a further increase in the proportion of staining (to 89%) (**Table 1**).

**Table 1:** Effect of piercing the seed coat on seed imbibition rate and on staining with triphenyl tetrazolium chloride (TTC), for four grass species.

			Staining with TTC (%)		
Species	Treatment	Imbibition (%)	No staining	Slight staining	Strong staining
*Elymus nutans*	Intact seeds	67	83	17	0
	Pierced seeds	77	5	1	94
*Lolium perenne*	Intact seeds	65	100	0	0
	Pierced seeds	70	4	12	84
*Leymus chinensis*	Intact seeds	62	91	9	0
	Pierced seeds	89	9	2	89
*Avena sativa*	Intact seeds	40	5	16	79
	Pierced seeds	56	6	4	89

### Relationships between EC, GP, and Field Emergence

**FIGURE 1:**
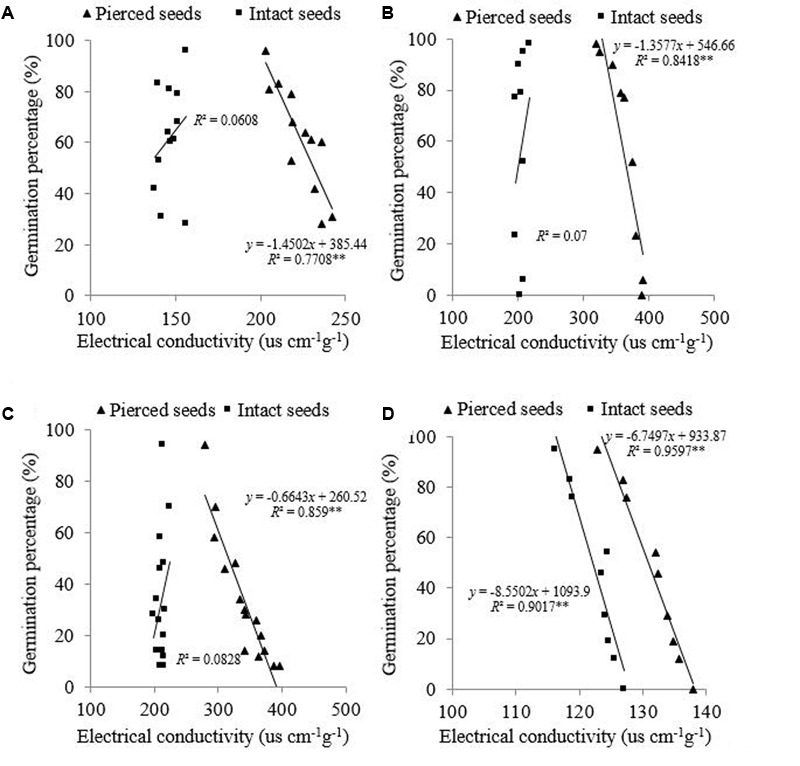
Relationship between electrical conductivity (EC) and germination percentage (GP) for **(A)** *Elymus nutans* Griseb., **(B)** *Lolium perenne* L., **(C)** *Leymus chinensis* (Trin.) Tzvel., **(D)** *Avena sativa* L. ^∗∗^*P* < 0.01.

The relationship between EC and GP was analyzed in both intact and pierced seeds following controlled deterioration treatment. In intact seeds of the three species that possessed a semipermeable layer (*E. nutans, Lolium perenne*, and *Leymus chinensis*), there was no correlation (*P* > 0.05) between EC and GP (*R*^2^ = 0.0608, 0.0700, and 0.0828, respectively) (**Figures 1A–C**). In pierced seeds of these species, however, EC showed a significant negative correlation (*P* < 0.01) with GP (*R*^2^ = 0.7708, 0.8414, and 0.859, respectively). In *A. sativa*, on the other hand, both intact and pierced seeds showed a significant negative correlation between EC and GP (*P* < 0.01; *R*^2^ = 0.9071 and 0.9597, respectively) (**Figure 1D**).

**FIGURE 2:**
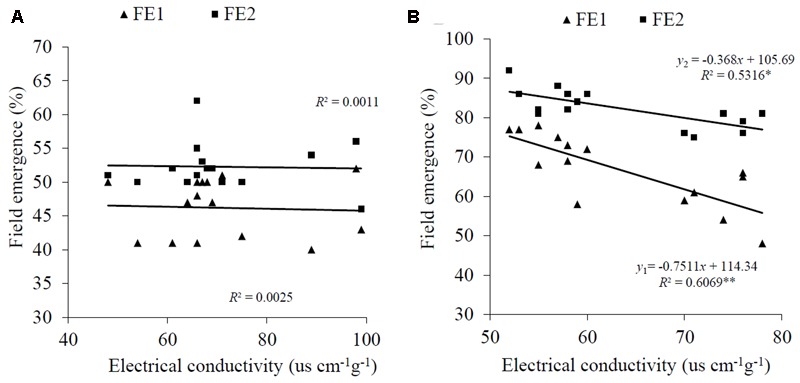
Relationship between EC and field emergence (FE) for **(A)** *E. nutans* Griseb. and **(B)** *A. sativa* L. FE1 is the estimate of FE for the first sowing time, and FE2 is the estimate for the second. ^∗^*P* < 0.05; ^∗∗^*P* < 0.01.

These results indicated that EC reflects seed germinability under laboratory conditions in *A. sativa*, but not in the other three species. This finding was borne out by a field experiment which showed that the EC value for intact *A. sativa* seed lots was negatively correlated with field emergence at the two sowing dates [*R*^2^ = 0.6069 (*P* < 0.01) and 0.5316 (*P* < 0.05), respectively] (**Figure 2B**). However, there was no correlation between EC and field emergence in *E. nutans* at either date (*R*^2^ = 0.0025, 0.0011; *P* > 0.05, respectively) (**Figure 2A**).

### Optical Microscopy of Seed Coat and Endosperm

**FIGURE 3:**
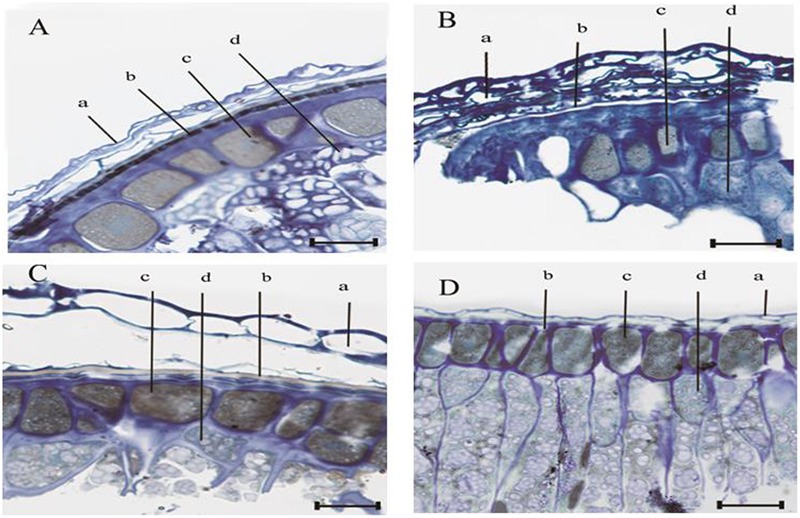
Light micrograph of the seed coat adjacent to the endosperm, for four grasses. **(A)** *E. nutans* Griseb., **(B)** *Lolium perenne* L., **(C)** *Leymus chinensis* (Trin.) Tzvel., **(D)** *A. sativa* L. Scale bar = 25 μm; ‘a’ denotes the pericarp; ‘b’ denotes the testa; ‘c’ denotes the aleurone layer; and ‘d’ denotes the endosperm.

The anatomy of the seed coat and endosperm for *E. nutans, Lolium perenne, Leymus chinensis*, and *A. sativa* is shown in **Figure 3**. In all four species, the structure of the seed coat adjacent to the endosperm was consistent with the basic characteristics of grass caryopsis, i.e., from the outside to the inside-pericarp, seed coat, aleurone layer, and starch. Moreover, the aleurone layer was in each case distinct and was stained positively with toluidine blue; however, the pattern of staining differed among species (**Figures 3A–D**). Thus, in *E. nutans* seeds, a black band was present between the pericarp and the aleurone layer (**Figure 3A**) and in *Lolium perenne* and *Leymus chinensis* seeds a white band was observable (**Figures 3B,C**); however, no band was observed in *A. sativa* (**Figure 3D**). It was deduced that this band represented the semipermeable layer and hence it was examined further by TEM and EDX.

### Determination of the Presence and Location of the Semipermeable Layer by TEM and EDX

**FIGURE 4:**
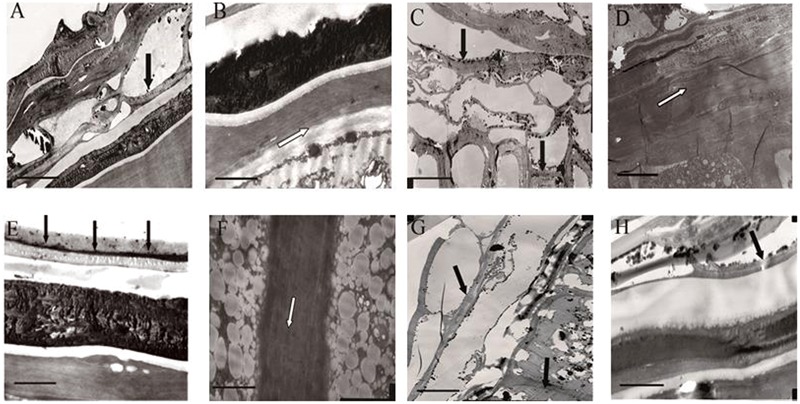
Transmission electron micrograph (TEM) of the seed coat and endosperm cell membrane, for four grasses. *E. nutans* Griseb.: **(A)** Pericarp and testa, **(B)** Testa and aleurone layer; *Lolium perenne* L.: **(C)** Pericarp, **(D)** Seed coat and aleurone layer; *Leymus chinensis* (Trin.) Tzvel.: **(E)** Pericarp and testa, **(F)** Aleurone layer cell membrane; *A. sativa* L.: **(G)** Pericarp testa and aleurone layer, **(H)** Testa and aleurone layer. The scale bar for **A,C,E,G** is 10 μm, and for **B,D,F,H** is 2 μm. Black dots are lanthanum granules, as indicated by black arrows.

**FIGURE 5:**
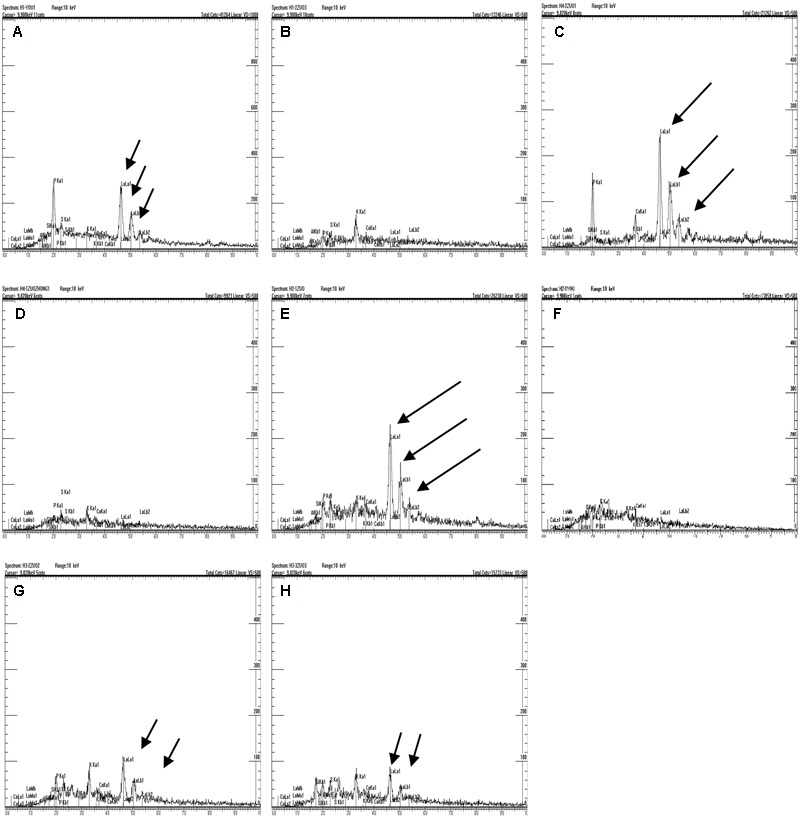
Analysis of the semipermeable layer using energy-dispersive X-ray spectroscopy (EDX). Black arrows indicate the lanthanum peak. *E. nutans* Griseb.: **(A)** Testa, **(B)** endosperm; *Lolium perenne* L.: **(C)** Testa, **(D)** endosperm; *Leymus chinensis* (Trin.) Tzvel.: **(E)** Testa, **(F)** Endosperm; *A. sativa* L.: **(G)** Testa, **(H)** Endosperm.

It was presumed that the semipermeable layer was the barrier that prevented lanthanum from penetrating further into the seed. As shown in **Figure 4** (in which the black arrow indicates the location of lanthanum accumulation, whereas the white arrow indicates absence of lanthanum), clear lanthanum deposits were found in the pericarp and the testa (**Figures 4A,C,E,G**). Analysis of the internal parts of the seeds of *E. nutans, Leymus chinensis*, and *Lolium perenne* showed no lanthanum (**Figures 4B,D,F**), suggesting that the semipermeable layer (the band described above) prevented lanthanum penetration. However, lanthanum was observed in the aleurone layer of *A. sativa* seeds (**Figure 4H**), suggesting that in this species no specific structure prevented lanthanum penetration. EDX analysis of the band showed that, whereas lanthanum was present in relatively large quantities in the seed coats of all the species (**Figures 5A,C,E,G**), there was no lanthanum in the endosperm of seeds of *E. nutans, Lolium perenne*, and *Leymus chinensis* (**Figures 5B,D,F**); however, it was present in the endosperm of *A. sativa* seeds (**Figure 5H**). This indicated that lanthanum distribution was unrestricted in *A. sativa* seeds, consistent with the absence of a semipermeable layer in this species.

## Discussion

Seed vigor declines during seed storage on account of a series of changes, such as the accumulation of reactive oxygen species, lipid peroxidation, loss of cellular membrane integrity, enzyme inactivation, weak energy metabolism, and DNA degradation (Kibinza et al., 2006; Parkhey et al., 2012; Ventura et al., 2012; Xin et al., 2014; Yin et al., 2014; Kong et al., 2015; Ratajczak et al., 2015; Wu et al., 2017). Generally, as seeds age, membrane integrity decreases and exosmosis occurs, and the EC value increases, thus reflecting seed vigor (Matthews and Bradnock, 1967; Hampton and TeKrony, 1995; Wang et al., 2002; Wang, 2003).

The present study clearly showed that intact seeds of *E. nutans, Lolium perenne*, and *Leymus chinensis* exhibited little staining with TTC; and that in these species, no change in the EC value was observed during seed deterioration, although GP declined (**Figures 1A–C**). In contrast, in *A. sativa*, a high percentage of seeds showed TTC staining, and in intact seeds of this species EC values were significantly negatively correlated with GP (**Figure 1D**). This suggests that in *E. nutans, Lolium perenne*, and *Leymus chinensis*, but not in *A. sativa*, there is a strong barrier that prevents solute leakage. Moreover, optical microscopy clearly suggested the presence of a semipermeable layer in the seed coats of *E. nutans, Lolium perenne*, and *Leymus chinensis* (**Figures 3A–C**), but not in that of *A. sativa* (**Figure 3D**). This was further supported by TEM and EDX analyses of lanthanum distribution following lanthanum nitrate infiltration, which showed that the distribution in the first three species did not extend beyond the seed (**Figures 4B,D,E, 5B,D,F**), whereas in *A. sativa* lanthanum was distributed internally, in the endosperm (**Figures 4F, 5F**).

Interestingly, for all species, piercing the seed coat resulted in a significant negative correlation between EC and GP (**Figures 1A–D**); i.e., as the EC value increased, GP declined. As compared to the behavior of intact seeds, cracks in the seed coat enhance both solute uptake and solute leakage. There are several factors affect EC value during seed deterioration (Yi et al., 1994; Han and Mao, 2000; Wang, 2003), among which, the semipermeable layer plays an important role, particularly. Piercing the seed coat loss integrity of the semipermeable layer and hence of the capacity to restrict solute movement.

Overall, it is apparent that, by reducing solute movement, the presence of a semipermeable layer negates the value of EC measurements in testing for seed vigor, in line with previous reports for seeds of *Allium tuberosum, Allium cepa, Lycopersicon esculentum*, and *Capsicum annuum* (Beresniewicz et al., 1995b; Taylor et al., 1995), and *Sorghum sudanense* (Wang, 2003; Wang et al., 2004). When a semipermeable layer is absent, for example in *A. sativa*, the EC value for intact seeds reflects seed germinability and seedling performance (see **Figures 1D, 2B**); the same observation has been made for *Glycine max* (L.) Merr. (Matthews and Bradnock, 1967; Loeffler et al., 1988), *Brassica oleracea* (Beresniewicz et al., 1995b; Taylor et al., 1995; Matthews et al., 2009), and *F. arundinacea* (Han et al., 1995). The phenomena of semipermeability appear to be widespread in seeds of higher plants. Previous researchers suggested that a seed semipermeable layer was present in 500 species belonging to 40 families; however, it was not found in the Leguminosae and in certain genera of the Cistaceae and Cruciferae (cited by Kotowski, 1927). The present study on grass species has shown for the first time that *A. sativa* does not possess a semipermeable layer. Similar results have been found for *F. arundinacea* (data not published). Although the location of the semipermeable layer varies with species (Beresniewicz et al., 1995a; Yan and Wang, 2008; Zhou et al., 2013a), its function is the same, which is to protect the mature embryo from drought stress (Prutsch et al., 2000), and to prevent or restrict the exchange of solutes whilst specifically permitting water and gas exchange to occur (Beresniewicz et al., 1995a). For many grasses, a semipermeable layer consisting of a cutin or suberin membrane in the caryopsis integuments restricts solute transport through the seed coat (Shull, 1913; Simpson, 1990). The degree of impermeability of the semipermeable layer may be related to its chemical composition (Beresniewicz et al., 1995b). Although seed aging causes some damage to membranes, it does not affect the semipermeable layer (Beresniewicz et al., 1995a,b; Yan, 2008). Taken together, these observations are all consistent with the function of the semipermeable layer as a strong barrier to restrict solute movement.

The significant negative correlation between EC and FE observed for *A. sativa* revealed that EC measurement was a useful predictor of seedling emergence in this species and provided a convenient and rapid method for seed vigor assessment. In contrast, the EC value was poorly correlated with field emergence in *E. nutans* (**Figure 5A**), which is consistent with results for *Sorghum sudanense, Elymus sibiricus* L. (Wang et al., 2004), *Lolium perenne* (Ching and Schoolcraft, 1968; Bennett et al., 1998), *Lolium multiflorum* Lam. (Marshall and Naylor, 1985), and *Bromus biebersteinii* (Hall and Wiesner, 1990). However, after the seed coat had been pierced, the EC value clearly reflected seed germinability in *E. nutans, Lolium perenne*, and *Leymus chinensis* also: the higher the EC value, the lower the seed germinability (**Figures 4A–C**). However, most grass species possess a seed semipermeable layer, and therefore TTC and EC values fail to estimate seed quality. The present study demonstrates that piercing the seed coat increases seed coat permeability, and suggests that EC measurements may then reflect seed germinability. However, whether EC values determined following piercing of the seed coat can predict seedling emergence for grass seeds possessing a semipermeable layer will require additional research.

## Conclusion

A semipermeable layer is present in the seed coats of *E. nutans, Lolium perenne*, and *Leymus chinensis*. The inability of TTC staining or EC tests to assess seed quality in these three species is confirmed to be attributable to the presence of a semipermeable layer. In *A. sativa*, on the other hand, which lacks a semipermeable layer, TTC staining and EC measurement can be used as predictors of seed viability and vigor.

## Author Contributions

YL did part of the experiment and write the paper. XQH did another part of the experiment. YW provided the guidelines and reversed the manuscript several times and provided the funding. XWH made some comments in writing paper.

## Conflict of Interest Statement

The authors declare that the research was conducted in the absence of any commercial or financial relationships that could be construed as a potential conflict of interest.
